# Serodiagnostic antigens of *Clonorchis sinensis* identified and evaluated by high-throughput proteogenomics

**DOI:** 10.1371/journal.pntd.0008998

**Published:** 2020-12-28

**Authors:** Pyo Yun Cho, Ji-Yun Lee, Tae Im Kim, Jin-Ho Song, Sung-Jong Hong, Won Gi Yoo, Takafumi Tsuboi, Kwon-Soo Ha, Jae-Wan Jung, Satoru Takeo, Eun-Taek Han, Banchob Sripa, Sung-Tae Hong, Jong-Yil Chai, Ho-Woo Nam, Jhang Ho Pak, Tong-Soo Kim

**Affiliations:** 1 Department of Medical Environmental Biology, Chung-Ang University College of Medicine, Seoul, Republic of Korea; 2 Animal & Plant Utilization Team, Nakdonggang National Institute of Biological Resources, Sangju, Republic of Korea; 3 Department of Pharmacology, Chung-Ang University College of Medicine, Seoul, Republic of Korea; 4 Proteo-Science Center and Venture Business Laboratory, Ehime University, Matsuyama, Ehime, Japan; 5 Department of Molecular and Cellular Biochemistry, Kangwon National University School of Medicine, Chuncheon, Republic of Korea; 6 Division of Tropical Diseases and Parasitology, Department of Infectious Diseases, Faculty of Medicine, Kyorin University, Mitaka, Japan; 7 Department of Medical Environmental Biology and Tropical Medicine, Kangwon National University School of Medicine, Chuncheon, Republic of Korea; 8 Department of Pathology, Khon Kaen University Faculty of Medicine, Khon Kaen, Thailand; 9 Department of Tropical Medicine and Parasitology, Seoul National University School of Medicine, Seoul, Republic of Korea; 10 Institute of Parasitic Diseases, Korea Association for Health Promotion, Seoul, Republic of Korea; 11 Department of Parasitology, Catholic University School of Medicine, Seoul, Republic of Korea; 12 Department of Convergence Medicine, University of Ulsan College of Medicine and Asan Institute for Life Sciences, Asan Medical Center, Seoul, Republic of Korea; 13 Department of Tropical Medicine, Inha University School of Medicine, Incheon, Korea; National University of Ireland Galway, IRELAND

## Abstract

Clonorchiasis caused by *Clonorchis sinensis* is endemic in East Asia; approximately 15 million people have been infected thus far. To diagnose the infection, serodiagnostic tests with excellent functionality should be performed. First, 607 expressed sequence tags encoding polypeptides with a secretory signal were expressed into recombinant proteins using an in vitro translation system. By protein array-based screening using *C*. *sinensis*-infected sera, 18 antigen candidate proteins were selected and assayed for cross-reactivity against *Opisthorchis viverrini*-infected sera. Of the six antigenic proteins selected, four were synthesized on large scale in vitro and evaluated for antigenicity against the flukes-infected human sera using ELISA. CsAg17 antigen showed the highest sensitivity (77.1%) and specificity (71.2%). The sensitivity and specificity of the bacterially produced CsAg17-28GST fusion antigen was similar to those of CsAg17 antigen. CsAg17 antigen can be used to develop point-of-care serodiagnostic tests for clonorchiasis.

## Author summary

Human clonorchiasis is a parasitic disease caused by the Chinese liver fluke, *Clonorchis sinensis*. Humans are infected through eating raw freshwater fishes carrying *C*. *sinensis* metacercariae, the encysted larvae. They excyst in the duodenum, move into the liver via bile duct and grow to adult worms. Excretory-secretory products of the worm damage the liver causing various inflammatory pathological changes and may lead to bile duct cancer. Although there exists an anthelmintic choice praziquantel to kill the fluke, emphasis is placed on early diagnosis and prevention before the infection becomes disease. Microscopic stool examination is the standard diagnostic method but is cumbersome and time consuming. Blood serum antibodies from clonorchiasis patients could provide a simple and fast diagnosis. However, antibody detecting diagnostics developed so far have a low specificity and sensitivity. In the present study we selected 607 antigenic candidate proteins from the genomic database and synthesized them through an integrated high-throughput proteogenomic tools. We identified several antigenic proteins and evaluated their diagnostic potential for clonorchiasis. One of them, CsAg17, showed a high sensitivity and specificity. This antigen deserves development of point-of-care serodiagnostics for *C*. *sinensis* infections.

## Introduction

Clonorchiasis is an infectious disease caused by a liver fluke, *Clonorchis sinensis*, and is endemic in East Asian countries, including China, Korea, Russia, and Vietnam. More than 200 million people are at risk of *C*. *sinensis* infection worldwide, and 15 million people have been infected in these countries thus far [1,2]. In general, ingestion of raw or inadequately cooked freshwater fish carrying *C*. *sinensis* metacercariae causes clonorchiasis. The metacercariae excyst in the duodenum, migrate up along the bile chemotaxis and into the intrahepatic biliary duct, and then grow into adult worms [3–6]. *C*. *sinensis* infections induce pathologic changes in the biliary tree, resulting in inflammation, hyperplasia of the biliary epithelium, periductal fibrosis, cholangitis, and cholangiectasis. *C*. *sinensis* along with *Opisthorchis viverrini* has been classified as a group I biological carcinogen causing cholangiocarcinoma [7,8].

The standard diagnostic method for *C*. *sinensis* infection is microscopic examination to detect eggs in stool samples; techniques such as Kato–Katz cellophane smear and formalin-ether centrifugal sedimentation can be used [9]. However, the microscopic stool examination is cumbersome and time consuming and should be performed by well-trained experts who can differentiate *C*. *sinensis* eggs from those of minute intestinal trematodes such as *Metagonimus yokogawai* [10]. The stool microscopies have shortcomings: 1) low egg detectability for specimens of patients with low worm burden and those in low endemic areas [11], and 2) low sensitivity at early stage of infection since the *C*. *sinensis* eggs can be detected in human feces after 4 weeks after the initial infection [2].

Serodiagnostic methods have been employed for epidemiological surveys as they are more suitable for screening of patients infected with *C*. *sinensis* and for supplementing the diagnosis of individual patients. The antigens used in serodiagnostics are crude worm extracts or recombinant proteins of *C*. *sinensis* adults [12–16]. These diagnostics, however, have low specificity and low sensitivity. Antigenic proteins have been identified from the excretory-secretory products (ESP) of *C*. *sinensis* [17]. The enzyme-linked immunosorbent assay (ELISA) using ESP as the antigen are more sensitive and specific than those using crude antigen. However, obtaining sufficient amounts of ESP is labor intensive and their quality is inconsistent [18]. Recombinant proteins may help overcome the shortcomings of preparing ESP for ELISA.

As single antigens for serodiagnosis of human clonorchiasis, the defined antigenic proteins have high specificity, but low sensitivity, especially toward the patients with low worm burden [17]. Hence, researches have been focused to identify effective serodiagnostic antigens for clonorchiasis. To increase sensitivity of the defined single antigens, a cocktail of multiple antigens was exploited as an alternative formula [19]. Hence, the expansion of the antigen candidate repertoire is demanding. In the post-genomics era, genomic information has enabled researchers to identify immunogenic proteins containing signal peptides and B-cell epitopes using immunoinformatics [20].

A wheat germ cell-free protein synthesis system (WGCFS) is an in vitro expression system taking advantage of the translation machinery in the wheat germs. When used for eukaryotic gene expression, it showed a powerful functionality for the synthesis of recombinant proteins from a wide range of eukaryotic organisms. This system is amenable to high-throughput protein synthesis bypassing many time-consuming steps of conventional expression systems [21,22].

This study aimed to discover novel antigen candidate proteins from *C*. *sinensis* transcriptome and validate their serodiagnostic antigenicity using integrated high-throughput proteogenomic tools.

## Materials and methods

### Ethics statements

The study protocol involving serum specimens was approved by the Institutional Review Board of Chung-Ang University (approval number: CAU-1041078-201401-BR-001-02). All adult participants provided informed consent. All personal identifiers and patient information were delinked from the serum specimens.

All animal handling and experimental procedures were reviewed and approved by the Institutional Animal Care and Use Committee at Chung-Ang University (approval number: 2011–00052). This study strictly followed the national guidelines outlined by the Korean Laboratory Animal Act (no. KCDC-122-14-2A) of the Korean Centers for Disease Control and Prevention.

### Serum

The human sera of clonorchiasis, opisthorchiasis, or paragonimiasis were collected from the patients infected with *C*. *sinensis*, *O*. *viverrini*, or *Paragonimus westermani* respectively. Their infections were confirmed by microscopic stool examinations and/or serologic ELISA employing respective antigens. The normal control sera were collected from healthy individuals in the low endemic areas in Korea [2]. The healthy individuals were proved to be without helminthic infection by microscopic stool examinations and ELISA as described above.

### Selection of putative secretory antigenic polypeptides

The expressed sequence tag (EST) sequences containing a secretory signal peptide at N-terminus were selected from the *C*. *sinensis* transcriptome [23,24] using the signal-natural networks and signal-hidden Markov model program (http://www.cbs.dtu.dk/services/SignalP/) and SignalP v3 [25]. The plasmid DNAs of EST clones were extracted and cut using endonuclease double digestion and checked for cDNA inserts using agarose gel electrophoresis. The fidelity of the cDNA inserts, a secretory signal peptide and its cleavage site, was confirmed by sequencing.

### Recombinant protein production using WGCFS

The wheat germ cell-free in vitro translation constructs of target cDNAs were prepared by performing two-step polymerase chain reaction (PCR) with supplementing regulatory transcription and translation elements to their 5′-end [21]. The cDNA insert in each EST was PCR-amplified individually using the forward primer specific and covering the 1^st^ Met of the deduced polypeptide encoded by the cDNA. A histidine-tag was added to N-terminus of each polypeptide to purify the recombinant protein using anti-His antibody (S1 Fig). The amplicons of primary PCR showing a single band (S2A Fig) were used in the secondary PCR. With clones producing non-specific products, PCR components were consecutively optimized until a single-band amplicon was produced. Secondary PCR was performed on the primary PCR amplicons. Finally, all amplicons showed single bands (S2B Fig).

The amplicons of secondary PCR were used for in vitro transcription, and the putative antigenic polypeptides encoded in the EST clones were synthesized using WGCFS [26] (WEPRO1240 Expression kit, CellFree Science, Matsuyama, Ehime, Japan) according to the manufacturer’s instruction. The WGCFS was performed using a robotic protein synthesizer, GenDecoder (CellFree Science).

### Selection of recombinant antigenic candidate proteins based on protein arrays

The antigenicity of recombinant proteins synthesized by WGCFS was measured on anti-His antibody-coated protein array chips using the sera of patients with clonorchiasis (S3 Fig). The well-type penta-His antibody arrays (Qiagen, Hilden, Germany) were prepared on glass slides [27]. The in vitro translates were spotted in duplicate wells of the array and incubated for 2 h at 37°C. The array contained positive and negative control proteins expressed using WGCFS [27]. The clonorchiasis serum was a pool of 5 positive individuals, and the normal control serum was of 5 healthy individuals. In our lab, for routine diagnostic ELISA, clonorchiasis patients’ sera had been diluted 1:100. In the present study, however, we wanted to get enough number of positive EST clones for downstream assays. Thus, the two sera were diluted 50-fold with PBS. The diluted sera, 1 μl each, were applied to the wells of the array and incubated for 1 h at 37°C. Then, secondary antibody, Alexa Fluor 546 goat anti-human IgG (10 ng/μl, Invitrogen, Carlsbad, CA), was applied to the array and incubated for 1 h at 37°C [27].

Fluorescence of the spots was scanned with a fluorescence scanner (ScanArray Express, PerkinElmer, Boston, MA) and quantified using the fixed circle method using ScanArray Express software version 4.0. A cutoff value was arbitrarily set at a fluorescence intensity of 1,800, and 18 antigenic candidate clones were selected. We considered these clones were manageable for downstream assays in our capacity.

The antigenicity of recombinant antigenic candidate proteins was reevaluated on the protein arrays as described above. In this assay, human sera were obtained from five each of clonorchiasis and opisthorchiasis patients, and normal healthy people.

### In-fusion cloning of antigenic candidate clones and recombinant protein expression using WGCFS in large scale

The cDNAs of selected ESTs were cloned into the WGCFS expression vector, pEU-E01-His-TEVMCS-N2 (pEU, CellFree Science). The coding region of the selected EST clone was amplified using a pair of primers containing cDNA-specific pEU vector homologous and adapted endonuclease restriction sequences. The amplicons were deployed in 1% agarose gel and purified using a purification kit (Qiagen). The pEU vector was double digested with restriction enzymes *Eco*RV and *Bam*HI. The amplicons were subcloned into pEU vector using the In-Fusion Advantage PCR Cloning Kit (Clontech, Mountain View, CA). The positive clones were selected by performing colony PCR using gene-specific primers. When double digested with two restriction enzymes, all positive clones surrendered each of the cDNA fragment inserted. The fidelity of the cDNA inserts was double checked by sequencing.

The recombinant antigenic candidate proteins were synthesized in a large scale using WGCFS as described above. The transcription was initiated by adding 25 μg of the expression construct plasmid DNA to the reaction mix. The synthesized recombinant proteins were purified using Ni-NTA chromatography (Qiagen), electrophoresed in 12.5% SDS-PAGE gel, and visualized using Coomassie Brilliant Blue staining.

### Soluble antigen of *C*. *sinensis* adults

The *C*. *sinensis* metacercariae were collected from the second intermediate host, *Pseudorasbora parva*, caught in a stream in Korea, after artificial digestion of the fish [28]. They were orally administered to rabbits (New Zealand White, both genders: 2.2–2.4 kg; Koatech Inc., Gyeonggi-do, Korea). Adult *C*. *sinensis* were recovered from the rabbits after 3 months, homogenized in two volumes of PBS containing protease inhibitor (1× Complete Mini, Roche, Mannheim, Germany), and incubated at 4°C overnight. The homogenate was centrifuged at 20,000 g for 60 min at 4°C, and the supernatant was saved as crude antigen. The protein concentration was determined using the Bio-Rad Protein Assay Kit (Bio-Rad, Hercules, CA).

### Antigenicity assay on recombinant antigenic proteins by ELISA

The serological antigenicity of the recombinant antigenic proteins synthesized using WGCFS was evaluated by performing an ELISA. Each of the 96-well plates was coated with the recombinant proteins or crude antigen of *C*. *sinensis* (0.5 μg/well) and incubated at 4°C overnight. The wells were blocked with 3% bovine serum albumin in PBS for 1 h at 37°C. The sera of humans infected with *C*. *sinensis*, *O*. *viverrini*, and *P*. *westermani*, and those from healthy individuals were diluted 1:100, and 100 μl of the mixture was poured into each well. A goat anti-human IgG horseradish peroxidase-conjugated (Sigma-Aldrich, St. Louis, MO) antibody at 1:4,000 dilution was added. Then, 200 μl of *o*-phenylene diamine (Sigma-Aldrich) substrate solution in 2.5% H_2_O_2_ was added and reacted for 20 min at 37°C. Absorbance was measured at 490 nm using a microplate reader. The cutoff value was set at mean + 2 standard deviations (SD) of the normal controls.

The human sera from 83 clonorchiasis patients, 35 opisthorchiasis patients, and 14 paragonimiasis patients, and 40 normal controls were used. Sera were obtained from patients diagnosed with clonorchiasis and opisthorchiasis on microscopic stool examinations, and those diagnosed with paragonimiasis based on the serological and/or pathological findings. Sera from normal controls were collected from healthy individuals without helminthic infection.

### Production of recombinant antigenic fusion proteins in *Escherichia coli*

The cDNA coding regions of the antigenic proteins selected by ELISA were PCR amplified using gene-specific primers containing restriction endonuclease sequences at both ends. The PCR amplicons were electrophoresed in 1% agarose gel and purified using a purification kit. The PCR amplicons were ligated into TA-cloning vector pCR2.1-TOPO (Invitrogen) and transformed into *E*. *coli*. The insert-positive colonies were selected using colony PCR. The inserted cDNAs were cut using the restriction enzymes *Bam*HI and *Eco*RI (TaKaRa, Shiga, Japan) and subcloned into a bacterial expression plasmid vector, pRSET-Cs28GST. This plasmid vector was genetically modified to carry a tag protein *C*. *sinensis* 28-kDa glutathione S-transferase (Cs28GST) by replacing Sj2GST. The Cs28GST is an antioxidant enzyme and soluble cytosolic protein. The recombinant Cs28GST is a soluble serodiagnostic antigen with high specificity for human clonorchiasis [29]. After confirming the Cs28GST + CsAg# cDNAs by sequencing, the expression plasmid construct was transformed into *E*. *coli* BL21[DE3]pLysS (Novagen, Madison, WI). After induction, the recombinant fusion proteins were purified under native conditions from the bacterial lysate using glutathione chromatography [29]. The purified fusion protein was dialyzed against PBS and electrophoresed in 12.5% SDS-polyacrylamide gel.

### ELISA on antigenic fusion proteins produced in *E*. *coli*

The antigenicity of the recombinant fusion proteins was reevaluated using ELISA as described above. A cocktail of four recombinant proteins, each with equal amount, was coated into the 96-well plate. The human sera used were obtained from 46 clonorchiasis patients and 30 normal controls.

## Results

### ESTs/cDNAs encoding putative secretory antigenic proteins

A total of 793 ESTs encoding putative secretory proteins were selected. They consisted of 247 singlets and 546 contigs. The overlapping ESTs were removed, and 556 EST reads were selected. In addition, 71 ESTs prospected to encode antigens of *C*. *sinensis* were screened by analyzing the annotated data. Of them, 51 ESTs were selected after scrutinizing the published studies and National Center for Biotechnology Information. The selected antigenic candidate ESTs were confirmed based on the read composition of contig, annotation information, and open reading frame. In total, 607 ESTs were selected as antigenic protein candidates—556 secretory proteins and 51 antigenic proteins. The plasmid DNA of the ESTs were extracted from the bacterial EST cell stocks and checked for cDNA inserts using two restriction enzymes and agarose gel electrophoresis. The length of the cDNA inserts ranged from 0.3 kb to 2.5 kb.

### Protein production using WGCFS

A total of 607 target cDNA inserts were amplified using two-step PCR. When electrophoresed on agarose gels, some primary PCR amplicons appeared as a single band (S2A Fig), but others showed more than two non-specific bands. For clones producing non-specific products, the PCR components were consecutively optimized several times; finally, single-band amplicons were secured from all clones. To incorporate the transcription and translation elements, a secondary PCR was performed. The secondary PCR produced all single-band amplicons (S2B Fig).

The 607 PCR-amplified cDNAs underwent recombinant protein synthesis in vitro using WGCFS. Analyses of the reaction mixtures on SDS-PAGE gel and western blot using an antibody against 6×His-tag protein showed 383 recombinant proteins. The WGCFS system demonstrated a recombinant protein synthesis success rate of 63.1%.

### Antigenicity of recombinant proteins to clonorchiasis patients’ sera

**Fig 1:**
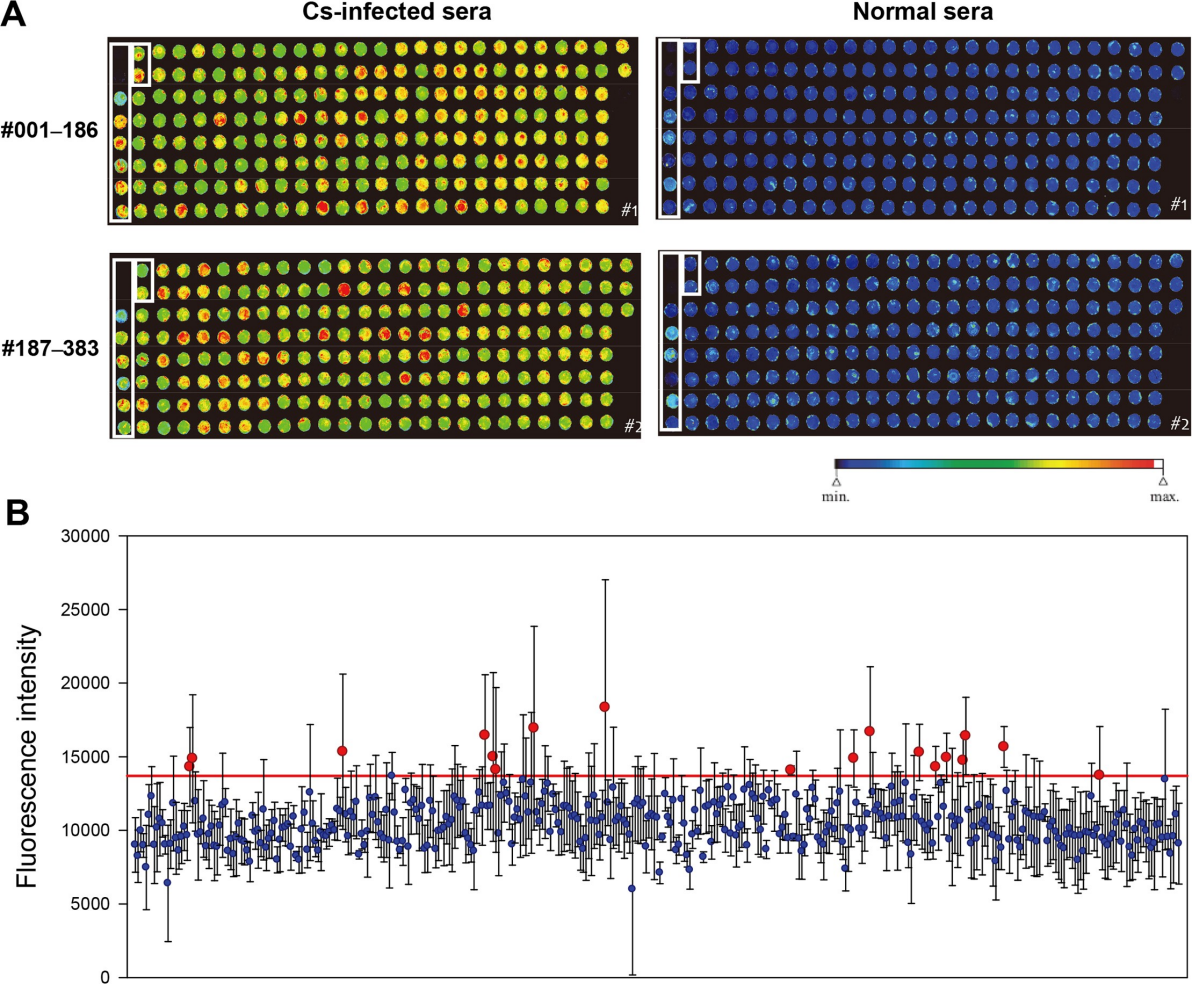
Antigenicity of recombinant proteins on protein array chips and selected candidates. (A) Fluorescent images of the recombinant proteins reacting to clonorchiasis patients’ sera and normal controls’ sera on protein array chips. The spots in boxes are positive and negative control proteins. Two spots top left are bovine serum albumin and wheat-germ extract itself, included as negative controls. Another 8 spots, top left to down, are recombinant *C*. *sinensis* 26 kDa glutathione-S-transferase (Cs26GST), Cs28GST, phosphoglycerate kinase, cathepsin F, and WGCFS translates of Cs26GST-1, Cs26GST-2, Cs28GST-1 and Cs28GST-2, included as positive controls. (B) Plot of fluorescence intensity (FI) of the recombinant proteins. Eighteen antigenic candidate proteins showing a higher FI were selected (above red line). Data are expressed as mean ± SD of triplicate measurements.

On the protein arrays, the 383 recombinant proteins were incubated with the sera of patients infected with *C*. *sinensis* and those of normal controls. Some proteins had high antigenicity with strong red fluorescence, while others had low antigenicity with blue fluorescence (Fig 1A). The fluorescence intensity (FI), which measures the antigen-antibody reaction, of sera from clonorchiasis patients was measured and normalized against the mean FI of the sera from normal controls. An arbitrary cutoff was drawn, and 18 antigenic candidate proteins of the higher titers were selected (Fig 1B).

### Antigenicity of antigenic candidate proteins

**Fig 2:**
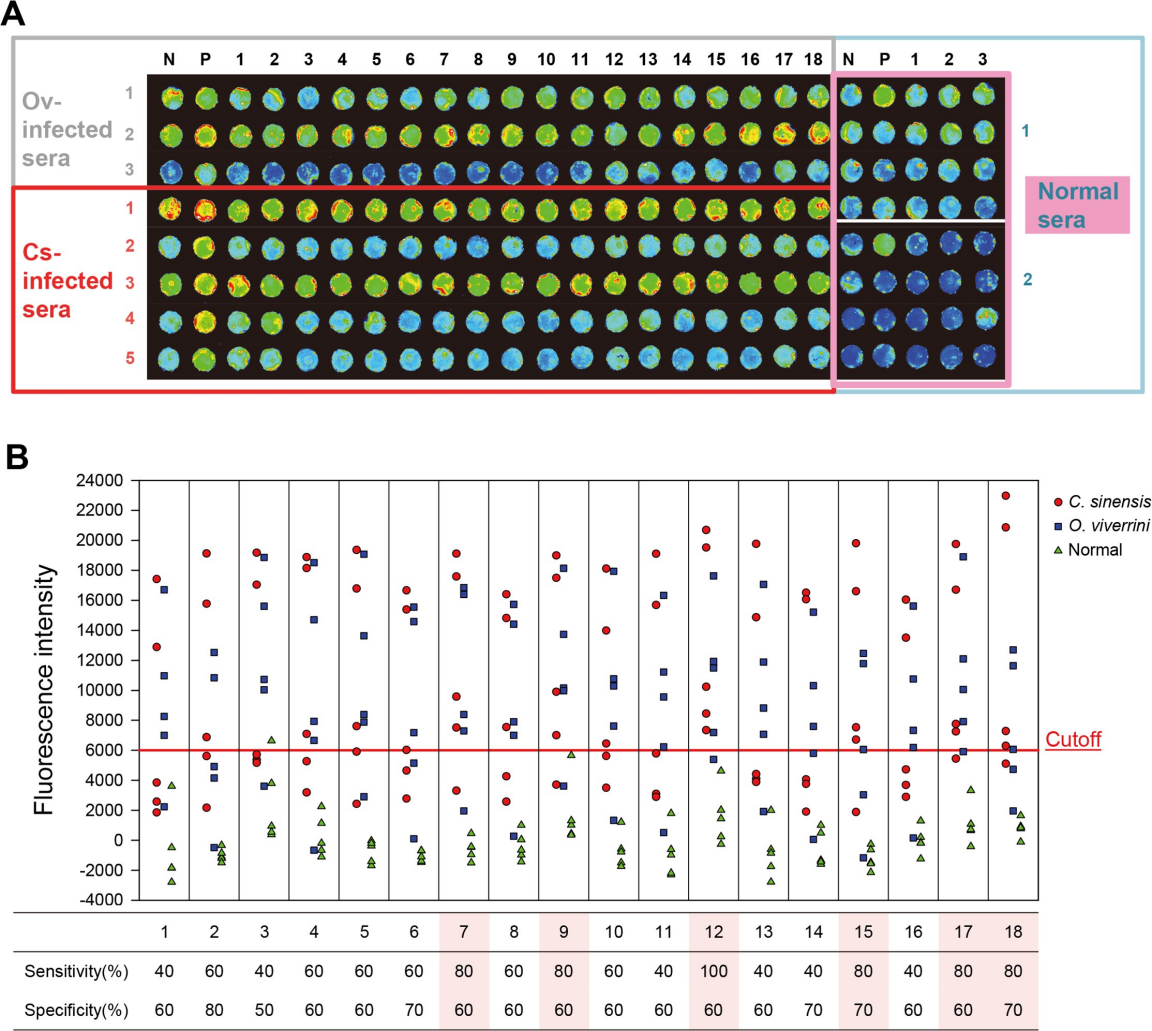
Serological antigenicity of antigenic candidate proteins. (A) A representative fluorescence image of protein array chip shows seroreactivity of the 18 antigenic candidate proteins toward liver fluke-infected and normal sera. N and P, negative and positive controls. In right boxes for normal sera, the 18 candidate proteins were arrayed 4×5 grid. (B) Fluorescence intensity and antigenicity of the candidate proteins. Spots indicate the mean of triplicate measurements. The proteins shaded in pink were selected as antigenic proteins. The sera used were obtained each from five *C*. *sinensis*- or *O*. *viverrini*-infected patients and normal controls.

The antigenicity of 18 recombinant antigenic candidate proteins was measured using the sera of clonorchiasis and opisthorchiasis patients on protein array chips (Fig 2A). A cutoff (mean + 2SD of normal sera) was set at FI 6,000. Six antigenic proteins showing a sensitivity of >80% and a specificity of >60% were selected (Fig 2B). These antigenic proteins (CsAg7, CsAg9, CsAg12, CsAg15, CsAg17, and CsAg18) were subjected to further serodiagnostic antigenicity assay.

### Serologic antigenicity of the antigenic proteins produced in large scale

To produce a large amount of recombinant proteins, cDNAs of the six antigenic proteins were sub-cloned into a plasmid vector, pEU-E01-His-TEVMCS-N2, establishing the expression plasmid constructs. The inserted cDNAs showed good fidelity on sequencing.

**Fig 3:**
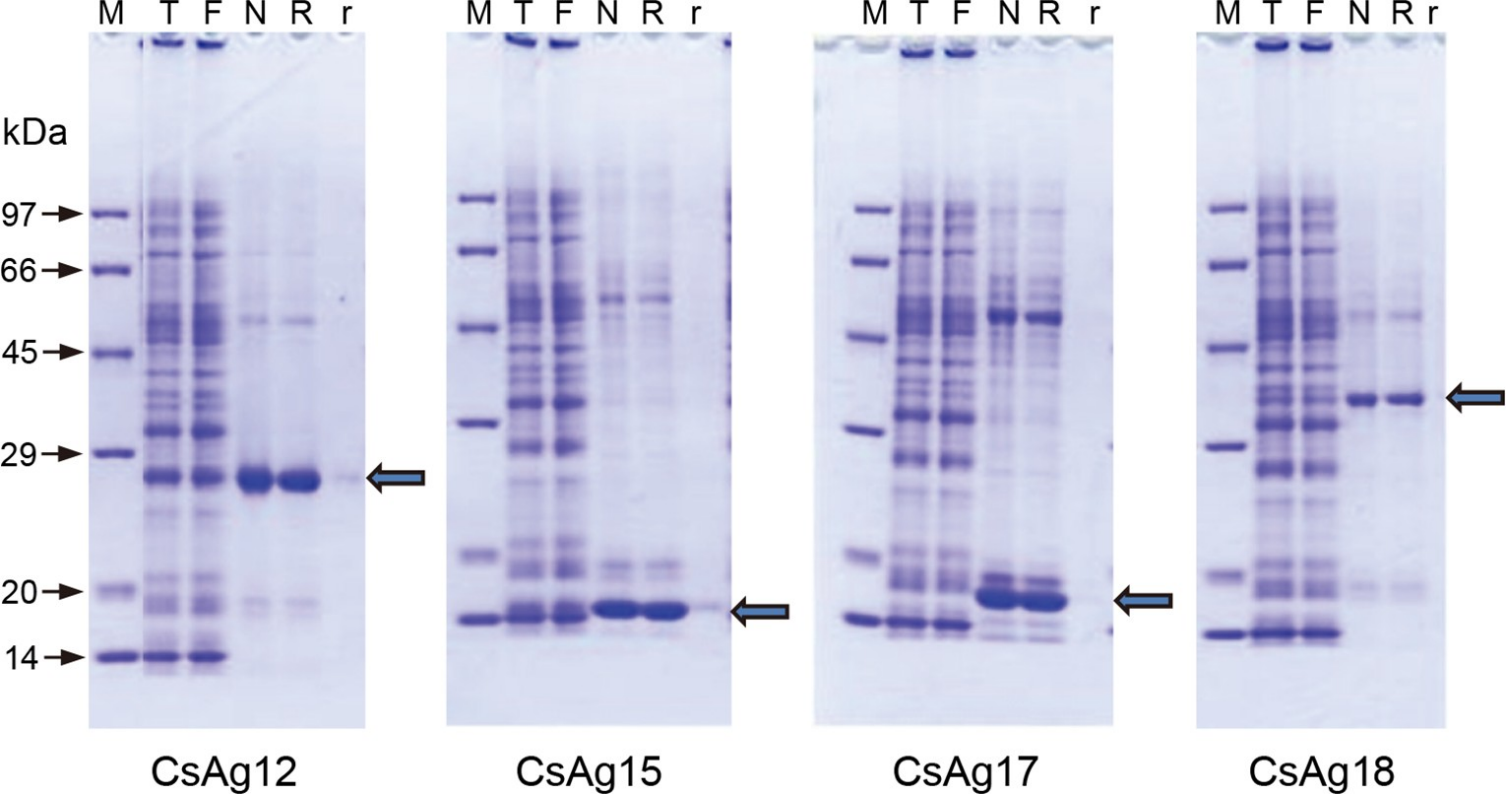
Recombinant antigenic proteins produced and purified in large scale using wheat germ cell-free protein synthesis system (WGCFS). The recombinant proteins were expressed using WGCFS, purified, and deployed on SDS-PAGE gel. The arrow indicates a purified recombinant protein having expected molecular mass. M, protein size marker; T, total translation mix; F, flow-through; N, non-reducing elution; R, reducing elution; r, resin after eluted.

The six antigenic EST clones were expressed on large scale using WGCFS and purified on Ni-NTA affinity column. Of them, CsAg12, CsAg15, CsAg17, and CsAg18 were produced in large amounts, enough to be evaluated for antigenicity. On SDS-PAGE gel, the recombinant antigenic proteins revealed each molecular mass plus His-tag (Fig 3, arrow).

**Fig 4:**
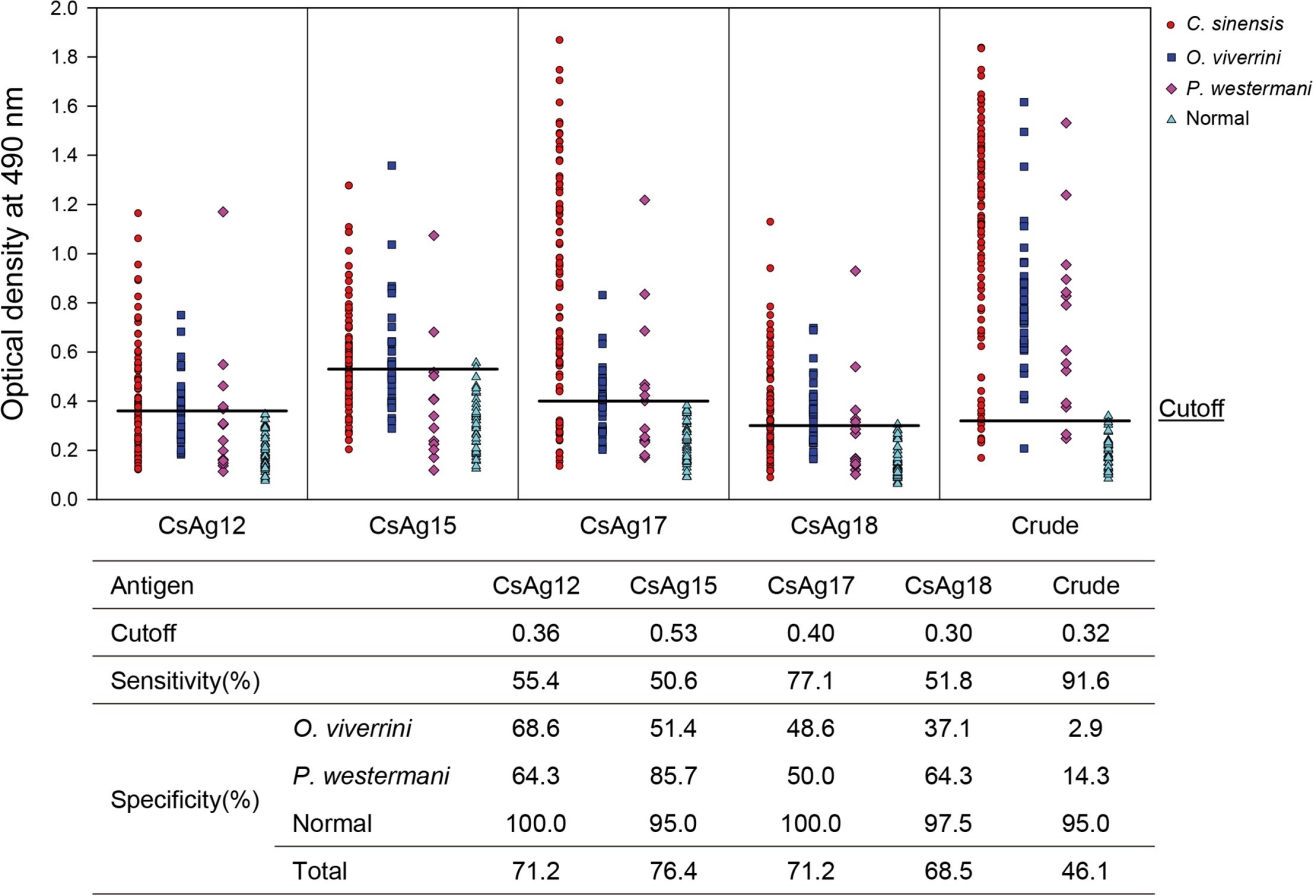
Serological antigenicity of recombinant antigenic proteins produced in large scale using wheat germ cell-free protein synthesis system. Antigenicity was measured by enzyme-linked immunosorbent assay against the fluke-infected human sera. The sera tested were obtained from 83 *C*. *sinensis*-, 35 *O*. *viverrini*-, and 14 *P*. *westermani*-infected patients, and 40 normal people.

The antigenic sensitivity and specificity of the four recombinant antigenic proteins were evaluated toward the sera of patients infected with *C*. *sinensis*, *O*. *viverrini*, and *P*. *westermani* using ELISA (Fig 4). Of the four CsAgs, CsAg17 showed the highest sensitivity (77.1%) and a high specificity (71.2%). It did not cross-react to sera of normal controls, but cross-reacted to the sera of the opisthorchiasis and paragonimiasis patients. The sensitivity of CsAg12, CsAg15, and CsAg18 ranged from 50.6% to 55.4%, with a specificity (68.5%–76.4%) similar to that of CsAg17. CsAg15 showed the highest specificity (76.4%) with the lowest cross-reactivity (14.3%) to the sera of patients with paragonimiasis, but it showed a low sensitivity (50.6%) (Fig 4). Crude antigen showed the highest sensitivity (91.6%), but the lowest specificity because of the higher cross-reaction to the sera of the fluke-infected patients. The full-length cDNA sequences of the four CsAgs were deposited to GenBank under the accession number MT62137, MT62138, MT62139, and MN381946.

### Antigenicity of fusion antigenic proteins produced using the *E*. *coli* system

The antigen-coding cDNAs were sub-cloned into the pRSET-Cs28GST expression vector. Inducing these expression plasmid constructs produced the fusion proteins (fCsAg#) of Cs28GST-antigenic protein. These fusion proteins were all overexpressed as soluble proteins in *E*. *coli* cytosol and purified to homogeneity by GSH-agarose column chromatography (S4 Fig). These antigenic fusion proteins were checked for serodiagnostic performance against clonorchiasis patients’ sera using ELISA.

**Fig 5:**
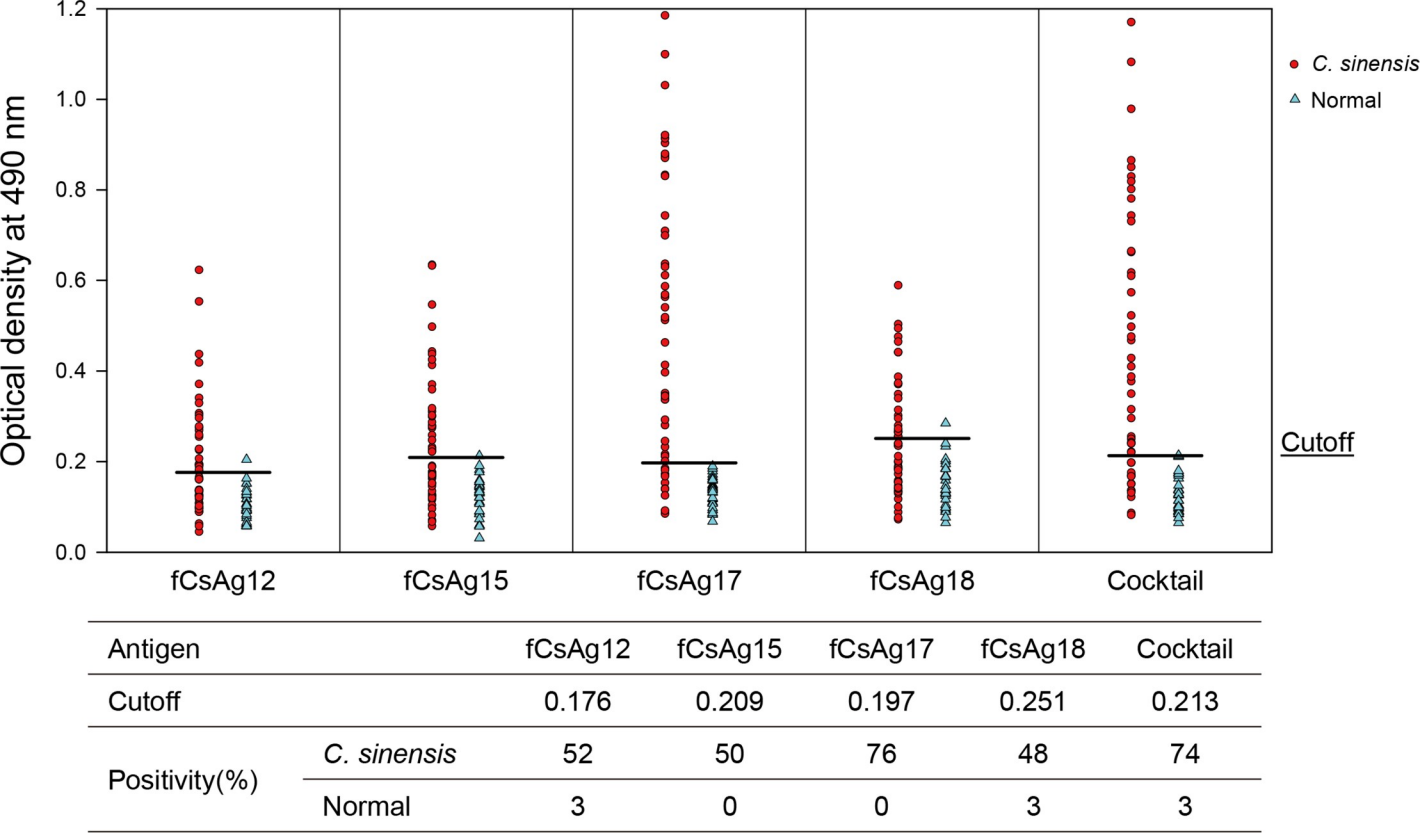
The serological antigenicity of fusion antigens (Cs28GST-CsAg#) produced in the *E*. *coli* system. A cocktail antigen was prepared by mixing equal amounts of four fusion antigens (fCsAgs). The antigenicity of the fCsAgs was measured by ELISA. The human sera used were obtained from 46 clonorchiasis patients and 30 normal people.

The fusion protein fCsAg17 showed the highest sensitivity (76%) to clonorchiasis sera without cross-reactivity to normal human sera. The other three fusion antigens—fCsAg12, fCsAg15, and fCsAg18—showed positive rates of 52%, 50%, and 48%, respectively, toward clonorchiasis sera (Fig 5). A cocktail antigen of all four fusion antigens showed high positive reactivity (74%) to clonorchiasis sera and cross-reacted to 3% of normal control sera.

## Discussion

Diagnosing *C*. *sinensis* infection classically involves microscopic detection of the parasite eggs from stool samples. To detect specific target genes [30,31], the PCR methods are sensitive but complicated in procedures, hindering field applications in the endemic areas. Serodiagnostic methods are useful for screening patients infected with *C*. *sinensis* and used to complement the stool microscopy. To develop efficient serodiagnostics for *C*. *sinensis* infections, much efforts have been made to obtain molecularly defined antigens with high sensitivity and specificity. The major task is to discover antigens that are better than crude antigens and excretory-secretory products (*Cs*ESPs), minimizing cross-reactivity to other trematode infections, increasing the sensitivity of the defined single antigens, and increasing the production of antigens [32]. The ELISA using crude extracts or *Cs*ESPs of adult worms is a reliable diagnostic tool for clonorchiasis. However, *Cs*ESPs show cross-reactivity to *P*. *westermani*- and *O*. *viverrini-*infected human sera [18]. It is difficult to produce a sufficient amount of *Cs*ESPs for serodiagnostic testing. Several recombinant proteins such as 7-kDa protein, CsEF-1α, 28-kDa cysteine protease, and 26-kDa and 28-kDa glutathione S-transferases of *C*. *sinensis* have shown relatively higher specificity than the *C*. *sinensis* crude extracts or ESPs for the serodiagnosis of clonorchiasis, but not sufficient sensitivity [17,33]. As alternatives, cocktails of multiple recombinant antigens and recombinant chimeric multi-epitope antigens were applied to enhance the antigenicity/sensitivity over single molecules [17–19].

The production of parasite recombinant proteins on large scale is a challenging task because traditional cell-based methods often yield insufficient amount of foreign proteins as soluble forms. With regard to the expression of recombinant proteins for serodiagnostic antigens, many different versions of cell-free protein synthesis systems have been developed to produce high-quality proteins in high-throughput systems [34]. In this regard, the wheat germ cell-free protein synthesis system is a good expression system for producing eukaryotic proteins in vitro and useful for high-throughput protein synthesis. Additionally, the wheat germ translates show a high solubility and translation yield [35].

The acquisition of an antigen with high sensitivity and specificity is essential for the serodiagnosis of clonorchiasis. In the current study, we performed in silico transcriptome-wide screening and in vitro high-throughput synthesis of recombinant proteins using WGCFS. As the first step, EST clones encoding a secretory signal peptide were mined in the *C*. *sinensis* transcriptome database using SignalP v3 with a neural network/hidden Markov model [25]. In the second step, the putative peptides were synthesized into recombinant proteins in vitro using a high-throughput cell-free system [26], and their antigenicity was determined using protein arrays [36] and ELISA. Finally, four diagnostic antigens were selected, with specificity and sensitivity higher than those of the crude antigens of *C*. *sinensis*. Among the four antigens, CsAg17 showed the highest sensitivity (77.1%) against human clonorchiasis sera and a much higher specificity (71.2%) compared with that of the crude antigen [18]. A cocktail mix of the four recombinant antigens showed almost the same antigenic reactivity as CsAg17 toward human clonorchiasis sera, suggesting that CsAg17 is dominating the antigenicity.

In immunogenicity assay, vaccination with CsAg17 cDNA and its B-cell epitope-derived protein exerted protective effects (64% and 69% reduction of worm burden, respectively) against *C*. *sinensis* infection [37]. The CsAg17 was predicted to have three linear and three conformational epitopes, and cytokine inducing peptides [38,39]. The epitopes and peptides induced production of immunoglobulins and cytokines, which orchestrated and exerted vaccination effects [37].

The bacterially produced fCsAg17 antigen could maintain its sensitivity and specificity toward the sera of clonorchiasis patients. When tagged to His-tag peptide, CsAg17 was overexpressed in *E*. *coli*, but majority of the recombinant protein remained as aggregate form [37]. As a cytosolic enzyme, Cs28GST fused to CsAg17 ensured solubility and overexpression in the prokaryote *E*. *coli*. The Cs28GST is an antigen of high specificity for clonorchiasis [29]. It could enhance sensitivity of the fCsAg17 antigen by detecting antibody in the serum which was not reactive to the CsAg17. The production of recombinant proteins using a bacterial system is economical and facilitative.

The CsAg17 was the most suitable antigen among the five antigenic proteins and its specificity and sensitivity was comparable to those of the *C*. *sinensis* soluble extract (crude antigen). Thus, CsAg17 antigen could be used to develop the point-of-care serodiagnostic tests and employed for mass screening and surveillance to estimate the *C*. *sinensis*-infected population. By employing the CsAg17-based serodiagnostics, the public health staffs can reduce working load for stool collection, preparation, and microscopic examination for the clonorchiasis control.

## Supporting information

S1 Fig Two-step PCR-synthesis of target cDNA. First, the target cDNA encoding a putative secretory antigenic polypeptide was amplified using a sense primer, S1 element + target cDNA-specific sequence covering the start codon and cleavage site, and an antisense primer, T3 promoter sequence of the plasmid vector DNA. Secondary PCR used two sense primers, SPU primer containing SP6 promoter and deSP6E01His-S1 primer containing 3′ terminus of SP6 promoter, E01 translational enhancer, His-tag and S1 sequences, and one antisense primer of T3 promoter the same as the primary PCR.
(TIF)

S2 Fig PCR amplicon examples of the two-step PCR for wheat germ cell-free protein synthesis system (WGCFS). The amplicons were produced as a single band in primary (A) and secondary (B) PCRs.
(TIF)

S3 Fig Schematic configuration of the anti-His antibody-coated protein array chips for analysis on antigenicity of recombinant proteins.
(TIF)

S4 Fig Production and purification of an antigenic fusion protein Cs28GST-CsAg17 (fCsAg17) in *E*. *coli*. An expression plasmid construct, pRSET-Cs28GST-CsAg17 was transformed into *E*. *coli* and induced by adding IPTG in culture medium. The fusion protein was purified on glutathione agarose column under native condition. kDa, molecular weight marker; Sol, soluble fraction; PT, pass-through.
(TIF)

## References

[1] Lun ZR, Gasser RB, Lai DH, Li AX, Zhu XQ, Yu XB, et al Clonorchiasis: a key foodborne zoonosis in China. Lancet Infect Dis. 2005; 5(1):31–41. 10.1016/S1473-3099(04)01252-6 15620559

[2] Hong ST, Fang Y. *Clonorchis sinensis* and clonorchiasis, an update. Parasitol Int. 2012; 61(1):17–24. 10.1016/j.parint.2011.06.007 21741496

[3] Kim TI, Yoo WG, Kwak BK, Seok JW, Hong SJ. Tracing of the bile-chemotactic migration of juvenile *Clonorchis sinensis* in rabbits by PET-CT. PLoS Negl Trop Dis. 2011; 5(12):e1414 10.1371/journal.pntd.0001414 22180795 PMC3236719

[4] Li S, Yoo WG, Song JH, Kim TI, Hong SJ. Bile acids drive chemotaxis of *Clonorchis sinensis* juveniles to the bile duct. PLoS Negl Trop Dis. 2018; 12(10):e0006818 10.1371/journal.pntd.0006818 30273341 PMC6181427

[5] Li S, Song JH, Kim TI, Yoo WG, Won MH, Dai F, et al Chemotactic migration of newly excysted juvenile *Clonorchis sinensis* is suppressed by neuro-antagonists. PLoS Negl Trop Dis. 2019; 13(8):e0007573 10.1371/journal.pntd.0007573 31408466 PMC6691982

[6] Dai F, Song JH, Hong YP, Bai X, Sohn WM, Hong SJ. Dopaminergic antagonists inhibit bile chemotaxis of adult *Clonorchis sinensis* and its egg production. PLoS Negl Trop Dis. 2020; 14(3):e0008220 10.1371/journal.pntd.0008220 32226018 PMC7145267

[7] Choi BI, Han JK, Hong ST, Lee KH. Clonorchiasis and cholangiocarcinoma: etiologic relationship and imaging diagnosis. Clin Microbiol Rev. 2004; 17(3):540–52. 10.1128/CMR.17.3.540-552.2004 15258092 PMC452546

[8] Bouvard V, Baan R, Straif K, Grosse Y, Secretan B, El Ghissassi F, et al A review of human carcinogens—Part B: biological agents. Lancet Oncol. 2009; 10(4):321–2. 10.1016/s1470-2045(09)70096-8 19350698

[9] Hong ST. Clonorchis sinensis In: Miliotis MD, Bier JW, editors. International handbook of foodborne pathogens. New York: Marcel Dekker, Inc.; 2003 p. 581–92.

[10] Lee SH, Hwang SW, Chai JY, Seo BS. Comparative morphology of eggs of heterophyids and *Clonorchis sinensis* causing human infections in Korea. Kisaengchunghak Chapchi. 1984; 22(2):171–80. 10.3347/kjp.1984.22.2.171 12891010

[11] Kim JH, Choi MH, Bae YM, Oh JK, Lim MK, Hong ST. Correlation between discharged worms and fecal egg counts in human clonorchiasis. PLoS Negl Trop Dis. 2011; 5(10):e1339 10.1371/journal.pntd.0001339 21991401 PMC3186755

[12] Yong TS, Yang HJ, Park SJ, Kim YK, Lee DH, Lee SM. Immunodiagnosis of clonorchiasis using a recombinant antigen. Korean J Parasitol. 1998; 36(3):183–90. 10.3347/kjp.1998.36.3.183 9755589 PMC2732929

[13] Na BK, Lee HJ, Cho SH, Lee HW, Cho JH, Kho WG, et al Expression of cysteine proteinase of *Clonorchis sinensis* and its use in serodiagnosis of clonorchiasis. J Parasitol. 2002; 88(5):1000–6. 10.1645/0022-3395(2002)088[1000:EOCPOC]2.0.CO;2 12435144

[14] Zhao QP, Moon SU, Lee HW, Na BK, Cho SY, Kong Y, et al Evaluation of *Clonorchis sinensis* recombinant 7-kilodalton antigen for serodiagnosis of clonorchiasis. Clin Diagn Lab Immunol. 2004; 11(4):814–7. 10.1128/CDLI.11.4.814-817.2004 15242967 PMC440603

[15] Ma C, Hu X, Hu F, Li Y, Chen X, Zhou Z, et al Molecular characterization and serodiagnosis analysis of a novel lysophospholipase from *Clonorchis sinensis*. Parasitol Res. 2007; 101(2):419–25. 10.1007/s00436-007-0481-3 17318582

[16] Shen C, Lee JA, Allam SR, Bae YM, Han ET, Takeo S, et al Serodiagnostic applicability of recombinant antigens of *Clonorchis sinensis* expressed by wheat germ cell-free protein synthesis system. Diagn Microbiol Infect Dis. 2009; 64(3):334–9. 10.1016/j.diagmicrobio.2009.03.003 19376673

[17] Kim TI, Na BK, Hong SJ. Functional genes and proteins of *Clonorchis sinensis*. Korean J Parasitol. 2009; 47 Suppl:S59–68. 10.3347/kjp.2009.47.S.S59 19885336 PMC2769219

[18] Choi MH, Park IC, Li S, Hong ST. Excretory-secretory antigen is better than crude antigen for the serodiagnosis of clonorchiasis by ELISA. Korean J Parasitol. 2003; 41(1):35–9. 10.3347/kjp.2003.41.1.35 12666728 PMC2717480

[19] Li S, Shin JG, Cho PY, Kim TI, Hong ST, Hong SJ. Multiple recombinant antigens of *Clonorchis sinensis* for serodiagnosis of human clonorchiasis. Parasitol Res. 2011; 108(5):1295–302. 10.1007/s00436-010-2179-1 21125293

[20] List C, Qi W, Maag E, Gottstein B, Muller N, Felger I. Serodiagnosis of Echinococcus spp. infection: explorative selection of diagnostic antigens by peptide microarray. PLoS Negl Trop Dis. 2010; 4(8):e771 10.1371/journal.pntd.0000771 20689813 PMC2914747

[21] Sawasaki T, Ogasawara T, Morishita R, Endo Y. A cell-free protein synthesis system for high-throughput proteomics. Proc Natl Acad Sci USA. 2002; 99(23):14652–7. 10.1073/pnas.232580399 12409616 PMC137474

[22] Yoo WG, Kim DW, Ju JW, Cho PY, Kim TI, Cho SH, et al Developmental transcriptomic features of the carcinogenic liver fluke, *Clonorchis sinensis*. PLoS Negl Trop Dis. 2011; 5(6):e1208 10.1371/journal.pntd.0001208 21738807 PMC3125140

[23] Chen JH, Jung JW, Wang Y, Ha KS, Lu F, Lim CS, et al Immunoproteomics profiling of blood stage *Plasmodium vivax* infection by high-throughput screening assays. J Proteome Res. 2010; 9(12):6479–89. 10.1021/pr100705g 20949973

[24] Kim DW, Yoo WG, Lee S, Lee MR, Kim YJ, Cho SH, et al ClonorESTdb: a comprehensive database for *Clonorchis sinensis* EST sequences. BMC Res Notes. 2014; 7:388 10.1186/1756-0500-7-388 24957044 PMC4094540

[25] Bendtsen JD, Nielsen H, von Heijne G, Brunak S. Improved prediction of signal peptides: SignalP 3.0. J Mol Biol. 2004; 340(4):783–95. 10.1016/j.jmb.2004.05.028 15223320

[26] Tsuboi T, Takeo S, Arumugam TU, Otsuki H, Torii M. The wheat germ cell-free protein synthesis system: a key tool for novel malaria vaccine candidate discovery. Acta Trop. 2010; 114(3):171–6. 10.1016/j.actatropica.2009.10.024 19913490

[27] Han JH, Li J, Wang B, Lee SK, Nyunt MH, Na S, et al Identification of immunodominant B-cell epitope regions of reticulocyte binding proteins in *Plasmodium vivax* by protein microarray based immunoscreening. Korean J Parasitol. 2015; 53(4):403–11. 10.3347/kjp.2015.53.4.403 26323838 PMC4566507

[28] Hong SJ, Seong KY, Sohn WM, Song KY. Molecular cloning and immunological characterization of phosphoglycerate kinase from *Clonorchis sinensis*. Mol Biochem Parasitol. 2000; 108(2):207–16. 10.1016/s0166-6851(00)00220-6 10838223

[29] Kang SY, Ahn IY, Park CY, Chung YB, Hong ST, Kong Y, et al *Clonorchis sinensis*: molecular cloning and characterization of 28-kDa glutathione S-transferase. Exp Parasitol. 2001; 97(4):186–95. 10.1006/expr.2001.4606 11384162

[30] Kaewkong W, Intapan PM, Sanpool O, Janwan P, Thanchomnang T, Laummaunwai P, et al Molecular differentiation of *Opisthorchis viverrini* and *Clonorchis sinensis* eggs by multiplex real-time PCR with high resolution melting analysis. Korean J Parasitol. 2013; 51(6):689–94. 10.3347/kjp.2013.51.6.689 24516275 PMC3916459

[31] Cho PY, Na BK, Choi KM, Kim JS, Cho SH, Lee WJ, et al Development of a polymerase chain reaction applicable to rapid and sensitive detection of *Clonorchis sinensis* eggs in human stool samples. Pathog Glob Health. 2013; 107(5):253–9. 10.1179/2047773213Y.0000000099 23916334 PMC4001454

[32] Kim JG, Ahn CS, Sripa B, Eom KS, Kang I, Sohn WM, et al *Clonorchis sinensis* omega-class glutathione transferases are reliable biomarkers for serodiagnosis of clonorchiasis and opisthorchiasis. Clin Microbiol Infect. 2019; 25(1):109 e1–e6. 10.1016/j.cmi.2018.03.042 29649604

[33] Cheng N, Xu XN, Zhou Y, Dong YT, Bao YF, Xu B, et al Cs1, a *Clonorchis sinensis*-derived serodiagnostic antigen containing tandem repeats and a signal peptide. PLoS Negl Trop Dis. 2018; 12(8):e0006683 10.1371/journal.pntd.0006683 30070987 PMC6091968

[34] Catherine C, Lee SW, Ju JW, Kim HC, Shin HI, Kim YJ, et al Cell-free expression and *in situ* immobilization of parasite proteins from *Clonorchis sinensis* for rapid identification of antigenic candidates. PLoS One. 2015; 10(11):e0143597 10.1371/journal.pone.0143597 26599101 PMC4657965

[35] Novikova IV, Sharma N, Moser T, Sontag R, Liu Y, Collazo MJ, et al Protein structural biology using cell-free platform from wheat germ. Adv Struct Chem Imaging. 2018; 4(1):13 10.1186/s40679-018-0062-9 30524935 PMC6244559

[36] Khan F, He M, Taussig MJ. Double-hexahistidine tag with high-affinity binding for protein immobilization, purification, and detection on Ni-nitrilotriacetic acid surfaces. Anal Chem. 2006; 78(9):3072–9. 10.1021/ac060184l 16642995

[37] Bai X, Song JH, Dai F, Lee JY, Hong SJ. *Clonorchis sinensis* secretory protein CsAg17 vaccine induces immune protection. Parasit Vectors. 2020; 13(1):215 10.1186/s13071-020-04083-5 32334611 PMC7183723

[38] Parker JM, Guo D, Hodges RS. New hydrophilicity scale derived from high-performance liquid chromatography peptide retention data: correlation of predicted surface residues with antigenicity and X-ray-derived accessible sites. Biochemistry. 1986; 25(19):5425–32. 10.1021/bi00367a013 2430611

[39] Jespersen MC, Peters B, Nielsen M, Marcatili P. BepiPred-2.0: improving sequence-based B-cell epitope prediction using conformational epitopes. Nucleic Acids Res. 2017; 45(W1):W24–W9. 10.1093/nar/gkx346 28472356 PMC5570230

