# Supplementary figures and images for: Serodiagnostic antigens of Clonorchis sinensis identified and evaluated by high-throughput proteogenomics
Source: PLoS Negl Trop Dis. 2020 Dec 28;14(12):e0008998. doi: 10.1371/journal.pntd.0008998 (PMC7793300; doi:10.1371/journal.pntd.0008998)

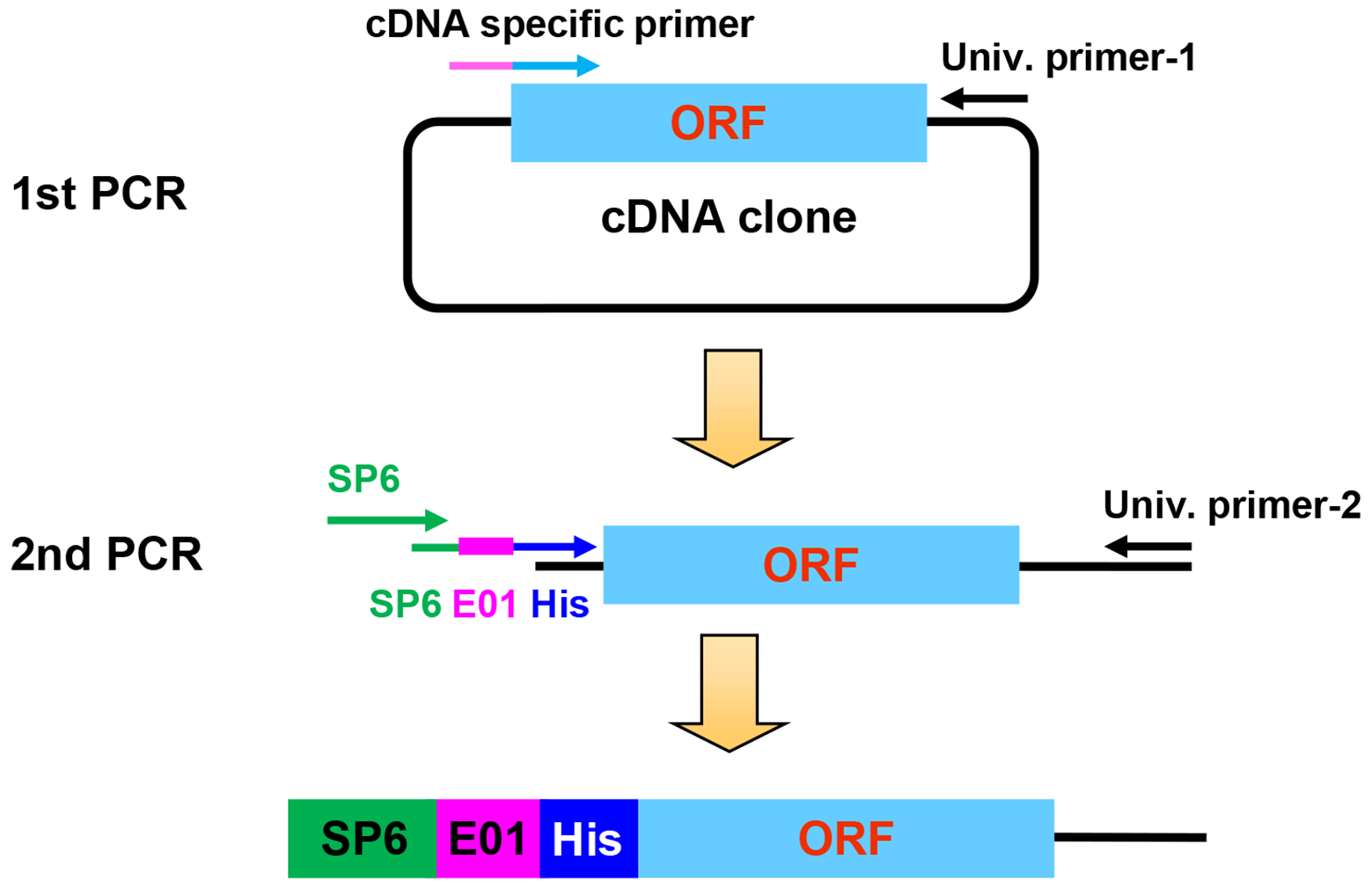

Supplement: S1 Fig — First, the target cDNA encoding a putative secretory antigenic polypeptide was amplified using a sense primer, S1 element + target cDNA-specific sequence covering the start codon and cleavage site, and an antisense primer, T3 promoter sequence of the plasmid vector DNA. Secondary PCR used two sense primers, SPU primer containing SP6 promoter and deSP6E01His-S1 primer containing 3′ terminus of SP6 promoter, E01 translational enhancer, His-tag and S1 sequences, and one antisense primer of T3 promoter the same as the primary PCR. (TIF) [file pntd.0008998.s001.tif]

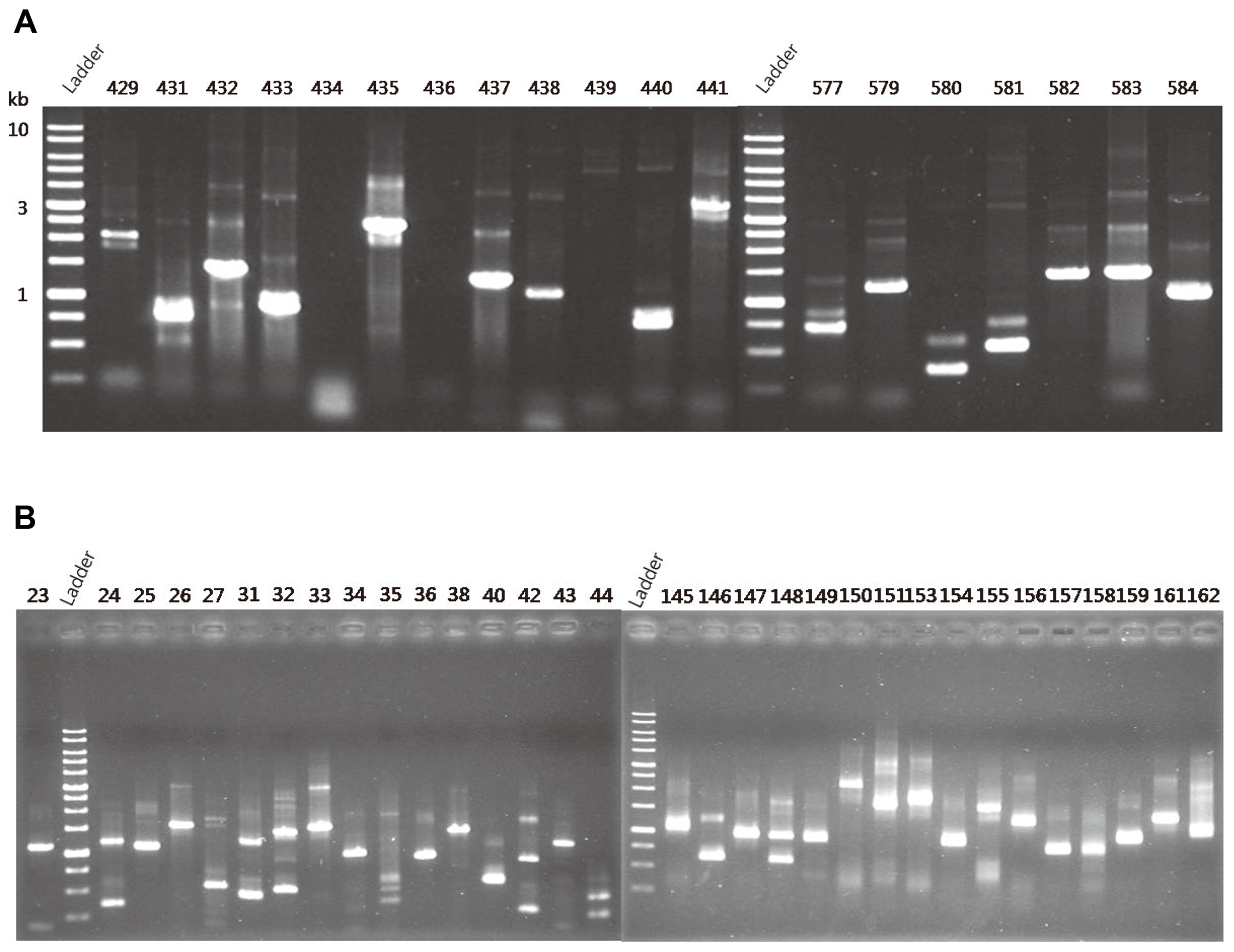

Supplement: S2 Fig — The amplicons were produced as a single band in primary (A) and secondary (B) PCRs. (TIF) [file pntd.0008998.s002.tif]

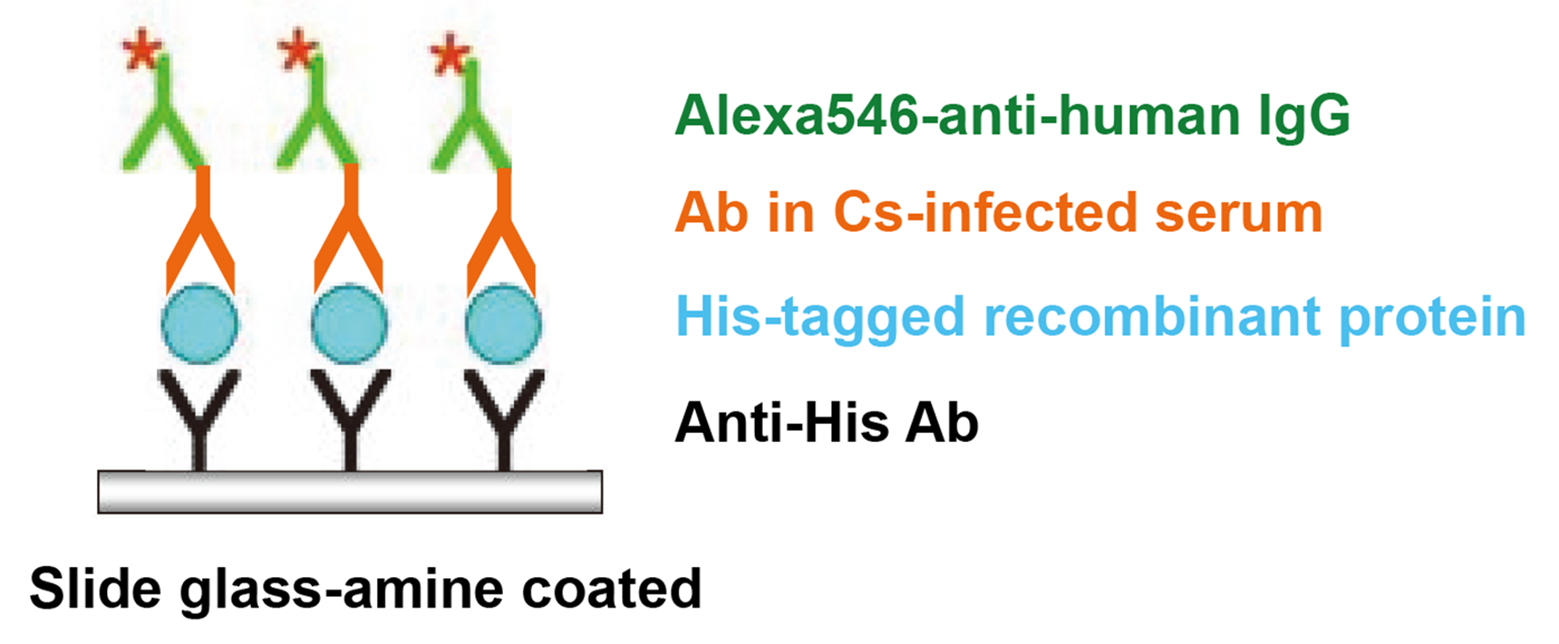

Supplement: S3 Fig — Schematic configuration of the anti-His antibody-coated protein array chips for analysis on antigenicity of recombinant proteins. (TIF) [file pntd.0008998.s003.tif]

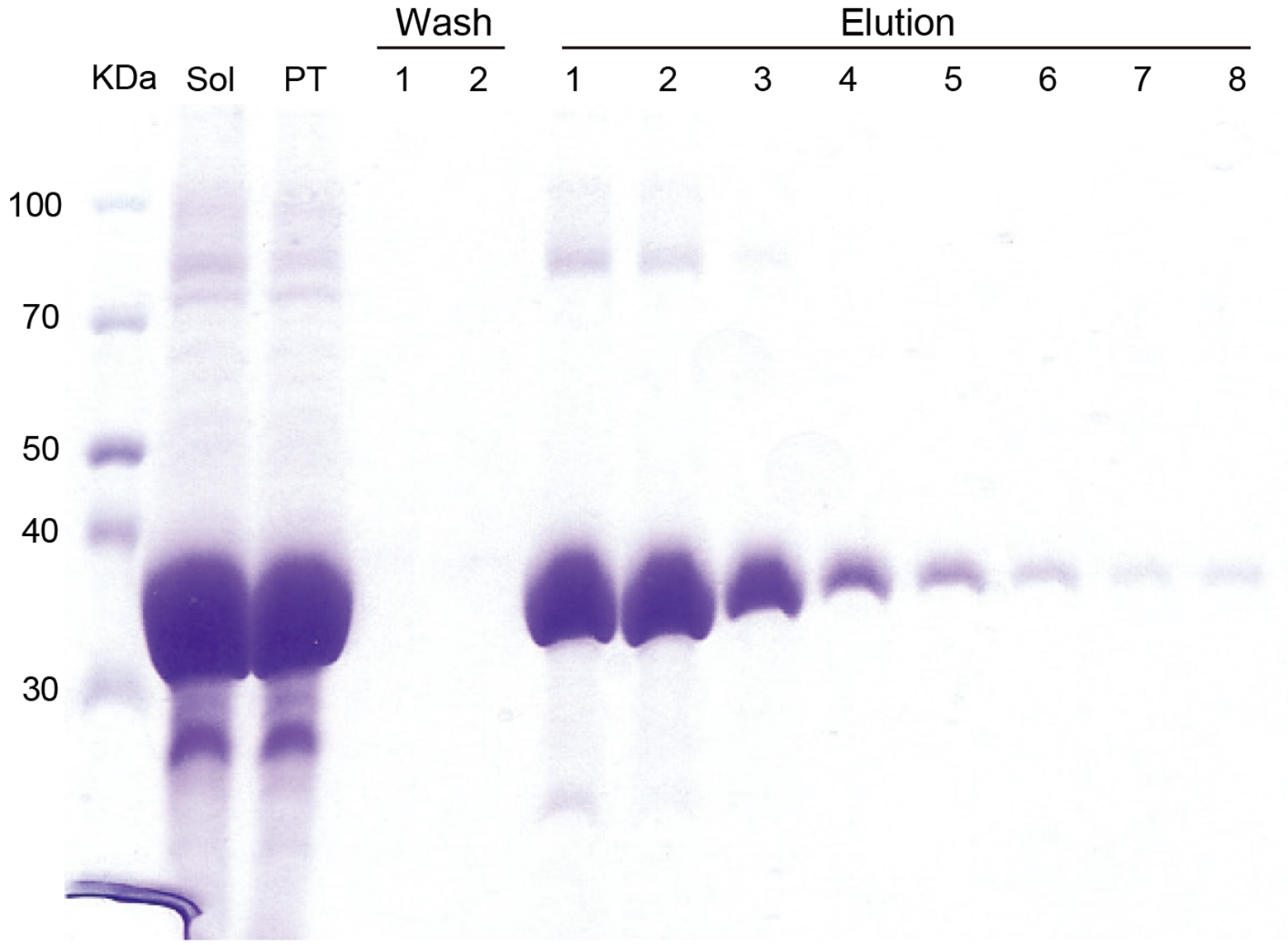

Supplement: S4 Fig — An expression plasmid construct, pRSET-Cs28GST-CsAg17 was transformed into E. coli and induced by adding IPTG in culture medium. The fusion protein was purified on glutathione agarose column under native condition. kDa, molecular weight marker; Sol, soluble fraction; PT, pass-through. (TIF) [file pntd.0008998.s004.tif]
